# Chromosomal Locations and Interactions of Four Loci Associated With Seed Coat Color in Watermelon

**DOI:** 10.3389/fpls.2019.00788

**Published:** 2019-06-25

**Authors:** Lucky Paudel, Josh Clevenger, Cecilia McGregor

**Affiliations:** ^1^ Institute for Plant Breeding, Genetics and Genomics, University of Georgia, Athens, GA, United States; ^2^ Department of Horticulture, University of Georgia, Athens, GA, United States

**Keywords:** *Citrullus lanatus*, *Citrullus amarus*, edible seed watermelon, seed coat color, QTL-seq, KASP™ assay, SNP markers, epistasis

## Abstract

Different species of edible seed watermelons (*Citrullus* spp.) are cultivated in Asia and Africa for their colorful nutritious seeds. Consumer preference varies for watermelon seed coat color. Therefore, it is an important consideration for watermelon breeders. In 1940s, a genetic model of four genes, *R*, *T*, *W* and *D*, was proposed to elucidate the inheritance of seed coat color in watermelon. In this study, we developed three segregating F_2_ populations: Sugar Baby (dotted black seed, *RRTTWW*) × plant introduction (PI) 482379 (green seed, *rrTTWW*), Charleston Gray (dotted black seed, *RRTTWW*) × PI 189225 (red seed, *rrttWW*), and Charleston Gray (dotted black seed, *RRTTWWdd*) × UGA147 (clump seed, *RRTTwwDD*) to re-examine the four-gene model and to map the four genes. In the dotted black × green population, the dotted black seed coat color (*R_*) is dominant to green seed coat color (*rr*). In the dotted black × red population, the dominant dotted black seed coat color and the recessive red seed coat color segregate for the *R* and *T* genes, where the *R* gene is dominantly epistatic to the *T* gene. However, the inheritance of the *T* locus did not fit the four-gene model, thus we named it *T^1^*. In the dotted black × clump population, the clump seed coat color and the dotted black seed coat color segregate for *W* and *D*, where *D* is recessively epistatic to *W*. The *R*, *T^1^*, *W*, and *D* loci were mapped on chromosomes 3, 5, 6, and 8, respectively, using QTL-seq and genotyping-by-sequencing (GBS). Kompetitive Allele Specific PCR (KASP™) assays and SNP markers linked to the four loci were developed to facilitate maker-assisted selection (MAS) for watermelon seed coat color.

## Introduction

Watermelon (*Citrullus lanatus*) is an annual, warm season vegetable crop which is grown throughout the tropical and sub-tropical regions of the world, predominantly for consumption of the sweet flesh. However, in many Asian and African countries, watermelons are instead cultivated for edible seeds. In China and India, most of the edible seed watermelons are from *C. lanatus* (Zhang, 1996; Mahla et al., 2014), whereas in West Africa, egusi watermelon, from the indigenous *C*. *mucosospermus* are extensively cultivated for edible seed (Oyolu, 1977; Gusmini et al., 2004). *C. colocynthis* is also cultivated as an edible seed watermelon in the Arabian peninsula and in India (Schafferman et al., 1998; Mahla et al., 2014). The land under edible seed watermelon production is increasing and the market has expanded from China, India and Africa to Europe and the Americas (Zhang, 1996; National Research Council, 2006; Mahla et al., 2014).

Seed coat color is an economically important trait because consumers prefer watermelon seeds with a specific color of seed. In China, seeds with red seed coat color, or seed with white center and a black margin are preferred (Zhang, 1996). Watermelon have a wide variety of seed coat colors ranging from flat black (solid black), dotted black (stipple black), tan, green, red, and clump to white (Poole et al., 1941; Poole, 1944). Flat black seeds have smooth, shiny, completely black seed coat, whereas dotted black seeds have a few to numerous black dots on an undercoat that can vary in color from whitish to red or even green. These black dots, which can usually be felt as protruding pins, provide dotted black seed coat a rough texture. Tan, green, and red seed coat have different shades of brown, green, and red color, respectively. Clump seed coat either have black pigment throughout the seed surface except on the narrow line inside the margin of the seed or have a white center, with a black rim on margin and/or two black spots on the hilum end (Poole et al., 1941). Description and naming of seed phenotypes has often been inconsistent among authors (Weetman, 1937; Poole et al., 1941; Sachan and Nath, 1976; Nath and Khandelwal, 1978) and for the sake of simplicity, we will use the phenotypic classification used by Poole et al. (1941).

Seed coat color is genetically controlled by a number of genes involving complex genetic interactions (Poole et al., 1941). The earliest attempt to study the inheritance of seed coat color was by Kanda (1931). He crossed flat black seeded watermelon with dotted black seeded watermelon and demonstrated that the flat black seed coat color is monogenically dominant to the dotted black seed coat color. Later, McKay (1936) developed two crosses: tan × red and green × red and showed that in each cross, the former phenotype was monogenically dominant to the latter phenotype. Weetman (1937) developed populations from three different crosses and showed that (1) the dotted black seed coat is monogenically dominant to the clump seed coat, and (2) different combinations of two genes produce clump and tan seed coat color. Poole et al. (1941) developed 40 different segregating populations and from the results they proposed a four-gene model controlling seed coat color in watermelon. According to this model, different combinations of three genes: *R*, *T*, and *W* with a modifier gene *D* (which only acts when the other three genes are in the dominant state) produce different seed coat colors, like flat black (*RTWD*), dotted black (*RTWd*), green (*rTW*), tan (*RtW*), clump (*RTw*), red (*rtW*), white tan-tip (*Rtw*), and white pink-tipped (*rtw*). This 1941 study was the last in-depth, large scale study on the genetics of watermelon seed coat color.

Next generation sequencing (NGS) technologies have made high-throughput sequencing less error-prone and very cost effective. As a result, NGS has become popular for the discovery of molecular markers throughout the genome (Varshney et al., 2009). GBS is a simple but highly scalable NGS-based genotyping model that can be used to genotype large populations and to identify thousands of genomic markers throughout the genome simultaneously (Elshire et al., 2011). GBS has been widely used to develop linkage maps and map quantitative trait loci (QTL) in several crops including watermelon (Lambel et al., 2014; Meru and McGregor, 2016a,b; Branham et al., 2017), zucchini (Montero-Pau et al., 2017), cucumber (Wang et al., 2018), pumpkin (Zhang et al., 2015), barley (Liu et al., 2014), pea (Boutet et al., 2016), rice (Bhatia et al., 2018), and alfalfa (Adhikari et al., 2018). Another relatively recent NGS-based technology is QTL-seq (Takagi et al., 2013). It combines bulk segregant analysis (Michelmore et al., 1991) with whole genome sequencing to identify QTL and to discover genetic markers necessary for MAS. One of the advantages of QTL-seq is that it does not require genotyping all the individuals in the population (Takagi et al., 2013). The first use of the QTL-seq approach in watermelon was to map a dwarfism locus on chromosome 7 (Dong et al., 2018). QTL-seq has been employed in several other crops like rice (Takagi et al., 2013), tomato (Illa-Berenguer et al., 2015), cucumber (Lu et al., 2014), chickpea (Das et al., 2015; Singh et al., 2016), and peanut (Clevenger et al., 2018).

Identification of the genomic regions associated with seed coat color is a crucial step in identifying candidate genes and in developing molecular markers for MAS. In this study, we used two interspecific and one intraspecific F_2_ populations segregating for different seed coat colors to (1) determine the location of the *R*, *T*, *W* and *D* loci responsible for seed coat color development in watermelon and (2) determine the interaction among these loci.

## Materials and Methods

### Plant Materials and Phenotyping

**Figure 1:**
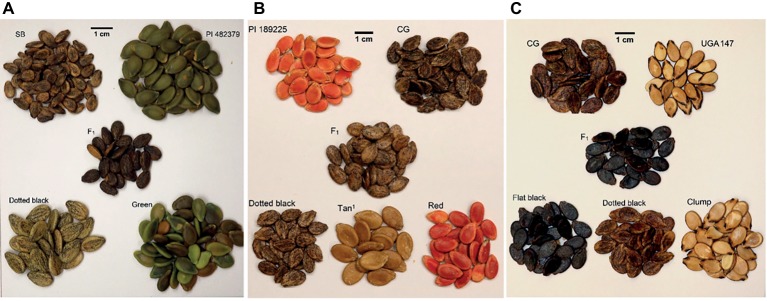
Seed coat color of parents, F_1_, and F_2_ progenies in **(A)** the dotted black × green population, **(B)** the dotted black × red population and **(C)** the dotted black × clump population. In the dotted black × green population **(A)**, seed of dotted black seeded Sugar Baby (SB), female parent, green seeded PI 482379, male parent, F_1_ and F_2_ individuals with dotted black and green phenotype. In the dotted black × red population **(B)**, seed of red seeded PI 189225, female parent, dotted black seeded Charleston Gray (CG), male parent, F_1_ and F_2_ individuals with dotted black, tan^1^ and red phenotype. In the dotted black × clump population **(C)**, seed of dotted black seeded Charleston Gray (CG), female parent, clump seeded UGA147, a selection from PI 169233, male parent, F_1_ and F_2_ progenies with flat black, dotted black and clump phenotype.

Three segregating F_2_ populations were used to identify the loci responsible for seed coat color development in watermelon. The dotted black × green F_2_ population (*n* = 128) was developed by crossing dotted black seeded Sugar Baby (*C. lanatus*) with green seeded PI 482379 (*C. amarus*) (Figure 1A). A dotted black × red F_2_ population (*n* = 96) was developed by crossing dotted black seeded Charleston Gray (*C. lanatus*) with red seeded PI 189225 (*C. amarus*) (Figure 1B). The dotted black × clump population (*n* = 178) used in this study was developed by Meru and McGregor (2016b) to map *Fusarium oxysporum* f. sp. *niveum* race 2 in sweet watermelon. This F_2_ population was produced by crossing dotted black seeded Charleston Gray (*C. lanatus*) with clump seeded UGA147 (*C. lanatus*), a selection from PI 169233 (Figure 1C).

The dotted black × green and dotted black × red parental cultivar/accession along with F_1_ plants and both F_2_ populations were sowed in the greenhouse on May 4, 2017 and transplanted in the field at the Durham horticulture farm (Watkinsville, GA) on May 30, 2017. The dotted black × green population was phenotyped in the field on August 24–25, 2017, and the dotted black × red population was phenotyped on September 7–10, 2017. Mature fruits from each plant were cut open and seeds were visually phenotyped. Dry seeds from the parental, F_1_ and F_2_ plants from the dotted black × clump population, grown in the greenhouse in 2012 and 2013, were visually phenotyped under daylight conditions. Seeds were harvested between 40 and 48 days after pollination. For all populations, seed were classified as dotted black if black dots or stipples were observed that were rough to the touch, irrespective of the undercoat color. This is in line with the phenotype as described by Poole et al. (1941) when developing the four gene model.

### Bulk Construction and DNA Isolation for QTL-seq

For QTL-seq of the dotted black × green population, a dotted black bulk (D-bulk) and a green bulk (G-bulk) were constructed by pooling equal amounts of DNA from 18 individuals of each phenotype. Similarly, for the QTL-seq of the dotted black × red population, a tan^1^ bulk (T-bulk) was developed by pooling equal amounts of DNA from 20 individuals with tan^1^ seed coat color and a red bulk (R-bulk) was developed by pooling equal amounts of DNA from 7 individuals with red seed coat color. Genomic DNA was extracted using the E. Z. N. A. Plant DNA kit (Omega Bio-Tek Inc., Norcross, GA) following the manufacturer’s protocol. DNA concentrations were measured using an Infinite M200Pro plate reader (Tecan, Group Ltd., Mannerdorf, Switzerland), and bulks were comprised from equal amounts of DNA from the selected individuals and sent to the HudsonAlpha Institute for Biotechnology (Huntsville, AL) for library preparation and 151 bp paired-end whole genome sequencing on the Illumina HiSeq X (Illumina, San Diego, CA).

### Analysis of NGS Data

A total of 168,613,320, 172,686,615, 124,764,246, and 154,206,455 reads for the D-bulk, G-bulk, T-bulk, and R-bulk were generated from NGS, respectively. The quality of the reads obtained from NGS was analyzed using FastQC (Andrews, 2010). To ensure that the average phred score for all of the base positions in all the reads was higher than 28, bases with a low phred score were trimmed on both ends for all the bulks as follows: the first seven bases of forward and reverse reads of all bulks, the last two bases of all forward reads, the last 41 bases of reverse reads of the D-bulk, the last 27 bases of reverse reads of the G-bulk, the last 31 bases of reverse reads of the T-bulk, and the last 27 bases of reverse reads of the R-bulk. The downstream analysis for all the bulks was the same. The trimmed reads were aligned against the 97103 watermelon genome (Guo et al., 2013) using default BWA and BWA MEM options (Li and Durbin, 2009). 165,489,026 (98.15%) reads from the D-bulk, 170,855,441(98.94%) reads from the G-bulk, 123,169,517 (98.72%) reads from the T-bulk, and 152,346,070 (98.79%) reads from the R-bulk were aligned with an average depth > 83×. SAM tools (Li et al., 2009) were used to sort, index, and calculate the genotype likelihood. BCF tools and a custom-made python script were used for SNP calling and filtering. A total of 4,953,800 SNPs was identified between the D-bulk and the G-bulk, and 3,401,764 SNPs were identified between the T-bulk and the R-bulk. The SNP-index, which is the proportion of reads harboring SNPs divided by the total number of reads for a genomic position, was calculated for every base in the genome for all bulks. The SNP-index of the G-bulk was subtracted from the SNP-index of the D-bulk to obtain a ΔSNP-index for the dotted black × green population, and the SNP-index of the R-bulk was subtracted from the SNP-index of the T-bulk to obtain a ΔSNP-index for the dotted black × red population. A custom python script was used to conduct sliding window analysis by averaging the ΔSNP-index within a 1 Mb window region with a 10 kb stepwise incremental. A permutation test was conducted to develop a null model assuming no QTL as explained by Takagi et al. (2013) and Clevenger et al. (2018). Thresholds for *p* < 0.05 and *p* < 0.01 were calculated for both population taking population size, number of individuals in each bulk, and read depth into account.

### DNA Extraction of F_2_ Populations and KASP™ Genotyping

Approximately 50 mg of leaf material from each individual of the dotted black × green and the dotted black × red parental cultivar/line, F_1_, and F_2_ populations were frozen in liquid nitrogen and ground using a TissueLyser II (QIAGEN, Hilden, Germany). DNA was extracted from leaf material using the King et al. (2014) extraction method with the following modifications. About 500 μl of extraction buffer mix [40% (v/v) 5 M NaCl and 60% (v/v) extraction buffer (200 mM Tris/HCl pH 7.5, 250 mM NaCl, 25 mM EDTA, 0.5% SDS)] was added on ground leaf material. Samples were vortexed, incubated for 30 min at 60°C, and centrifuged for 10 min at 3600 rpm. An equal amount of supernatant and isopropanol was mixed and centrifuged to obtain DNA pellets. The DNA pellets were washed with 70% alcohol, dried, and resuspended in 200 μl TE buffer.

To validate the association of significant peaks with seed coat color, SNPs identified through QTL-seq were converted into KASP™ assays (Table 1). Primers were designed using Primer3Plus (Untergasser et al., 2007), and PCR amplification was done using a S1000™ Thermo Cycler (Bio-Rad Laboratories, Inc., Hercules, CA). The 4 μl PCR reaction included 2 μl of 50–100 ng/μl genomic DNA, 1.96 μl 2× low rox KASP™ master mix (LGC Genomics LLC, Teddington, UK), and 0.06 μl primer mix for a final concentration of 0.81 μM. The PCR conditions for the KASP™ assays were set as follows: 95°C for 15 min, followed by 10 cycles of touch down PCR (95°C for 20 s, primer annealing temperature + 9°C for 25 s with 1°C decrease each cycle and 72°C for 15 s), then followed by 35 additional cycles (95°C for 10 s, primer annealing temp for 1 min, and 72°C for 15 s). PCR florescent end-point readings was done using an Infinite M200Pro plate reader (Tecan, Group Ltd.), and genotyping calls were carried out using KlusterCaller™ (LGC Genomics LLC). Individuals whose florescent end-point readings for markers were ambiguous were called missing data and were excluded from genotypic analysis. This caused discrepancies between the number of individuals that were phenotyped and genotyped.

**Table 1:** KASP™ assays used to test association between significant genomic regions, identified from QTL-seq, and the seed coat color phenotype in watermelon. The marker names indicate the chromosome number and physical position of the marker based on 97103 watermelon genome (Guo et al., 2013).

KASP assay	Primer type	Primer sequence (5′-3′)	Tm (°C)
UGA3_5820134	FAM	GAAGGTGACCAAGTTCATGCTTAGAGACACAAGAAAGTTGCAAAGG	61.1
	VIC	GAAGGTCGGAGTCAACGGATTTAGAGACACAAGAAAGTTGCAAAGT	61.1
	Reverse	TCATTTATTTCCCTCCTTAGCTTTCA	62.5
UGA5_4591722	FAM	GAAGGTGACCAAGTTCATGCTTTGTGAAATCAAAGATATGGACCAA	61.7
	VIC	GAAGGTCGGAGTCAACGGATTTTGTGAAATCAAAGATATGGACCAG	61.7
	Reverse	GAGTTACTTGAATTTGGAAAGGAAAGG	62.6

### Genotyping of Dotted Black × Clump and Construction of a Genetic Linkage Map

GBS of the dotted black × clump population is described in Meru and McGregor (2016b). The original 501 SNPs for the population was filtered using Joinmap 5.0 (Van Ooijen, 2006) for missing data (with up to 20% missing data) and segregation distortion (*p* < 0.0001). The remaining 230 SNPs were ordered using the regression mapping algorithm and grouped into linkage groups at LOD 5. A linkage map was generated using the Kosambi mapping function by converting recombination frequencies into map distances in centimorgan (cM).

## Results

### Phenotypic Segregation in the Dotted Black × Green Population and Mapping of the *R* Locus

In the dotted black × green population, F_1_ plants had seeds with dotted black seed coat color indicating that dotted black is dominant over the green (Figure 1A). Initially, it seemed that the F_2_ progeny included dotted black, green, and brown seed phenotypes. However, upon closer inspection, it was established that the green seed turned brown over time and this difference was due to maturity. Green and brown seed could be observed in fruit harvested from a single plant (Supplementary Figure S1, Figure 1A). This phenotype was classified as green to conform with Poole et al. (1941). The F_2_ progenies segregated at a ratio of 88 dotted black to 40 green seeded individuals. A chi-square goodness of fit test shows that the observed segregation ratio fits a 3:1 ratio ($\chi_{\left(0.05,1\right)}^{2}$ = 2.67, *p* = 0.10). This result confirms the conclusion made by Poole (1944) that the dotted black (*R_*) seed coat color is monogenically dominant to the green seed coat color (*rr*).

**Figure 2:**
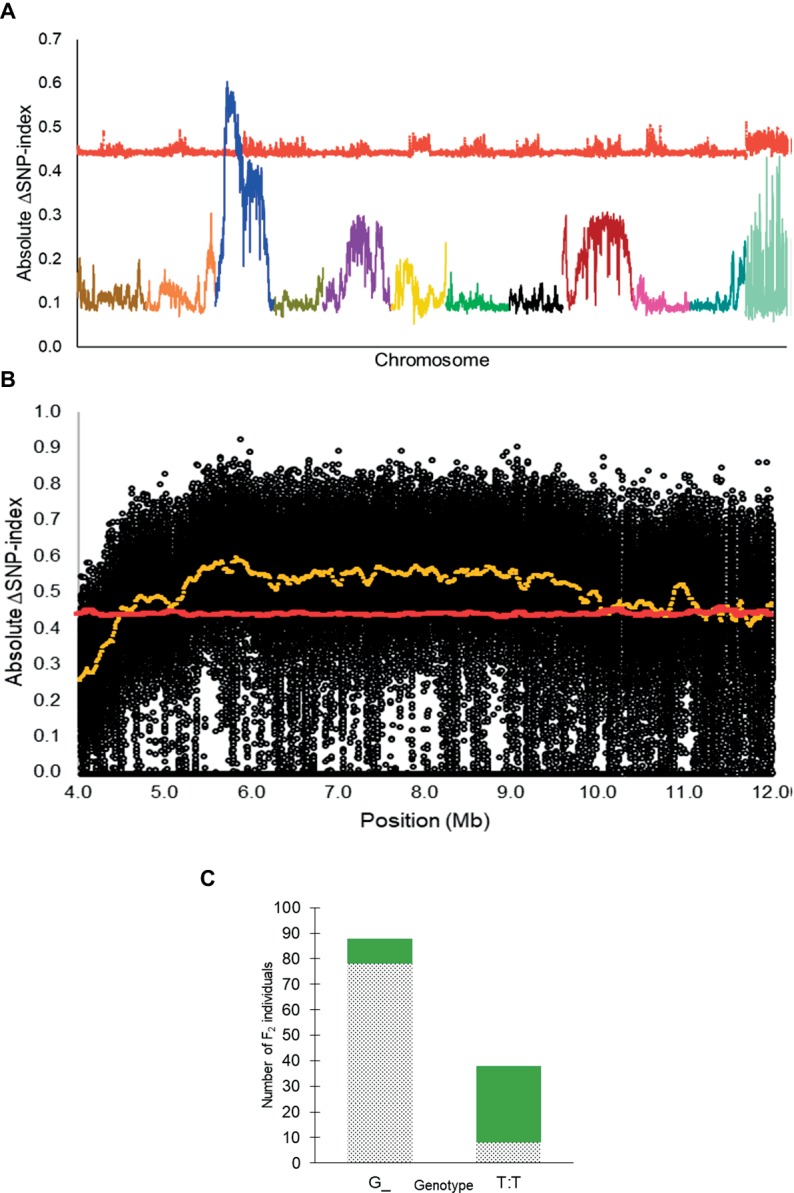
**(A)** Absolute ΔSNP-index of all chromosomes in the inter-specific dotted black × green F_2_ population developed from a cross between Sugar Baby (*C. lanatus*) and PI 482379 (*C. amarus*) plotted along with statistical confidence intervals under the null hypothesis of no QTL (*p* = 0.01) (red line). The *R* locus is mapped on chromosome 3. **(B)** Magnified view of *R* locus, a significant ΔSNP-index peak (yellow), along with absolute ΔSNP-index of SNPs (black circles) plotted against the SNP position. SNP positions are based on the 97103 watermelon genome (Guo et al., 2013). **(C)** Association of KASP™ marker UGA3_5820134 with seed coat color phenotype in the dotted black × green F_2_ population (*n* = 126). The dotted black and green sections in the graph indicate the number of F_2_ individuals with dotted black and green seed coat color, respectively.

From QTL-seq, a significant ΔSNP-index peak (*p* < 0.01) was identified from 4.48 to 12.98 Mb on chromosome 3 of the *C. lanatus* genome (Figures 2A,B). A KASP™ assay, UGA3_5820134 (Table 1), was designed for a SNP located near the highest peak [5,820,134 bp on chromosome 3 of 97103 reference genome (Guo et al., 2013)] to test the association between the significant peak and the phenotype. The assay was able to accurately predict the phenotype of 85.7% (*n* = 126) of individuals (Figure 2C), confirming the association of this region with the *R* locus.

### Phenotypic Segregation in the Dotted Black × Red Population and Mapping of the *T^1^* Locus

The F_1_ plants in the dotted black × red population have seeds with dotted black seed coat denoting that the dotted black seed coat is dominant over the red seed coat color (Figure 1B). The segregating F_2_ progenies had either dotted black, red or tannish (light shade of brown with yellowish tinge, similar to khaki) seed coat color. According to the four-gene model, F_2_ individuals in a dotted black × red population is expected to have either dotted black, tan, green or red seed coat color at a 9 dotted black (*R_T_*): 3 tan (*R_tt*): 3 green (*rrT_*): 1 red (*rrtt*) ratio. In the current study, no individuals with green seed color were observed in the dotted black × red population. The tannish seed coat color observed was different from the range of brown color (“dark Tuscany brown to cacao”) used to describe tan seed coat color by Poole et al. (1941). Therefore, we classified this phenotype as tan^1^. The F_2_ progenies segregated at the ratio of 67 dotted black: 22 tan^1^: 7 red which statistically corresponds to 12:3:1 ratio ($\chi_{\left(0.05,2\right)}^{2}$ = 1.40, *p* = 0.49) and indicates dominant epistasis.

**Figure 3:**
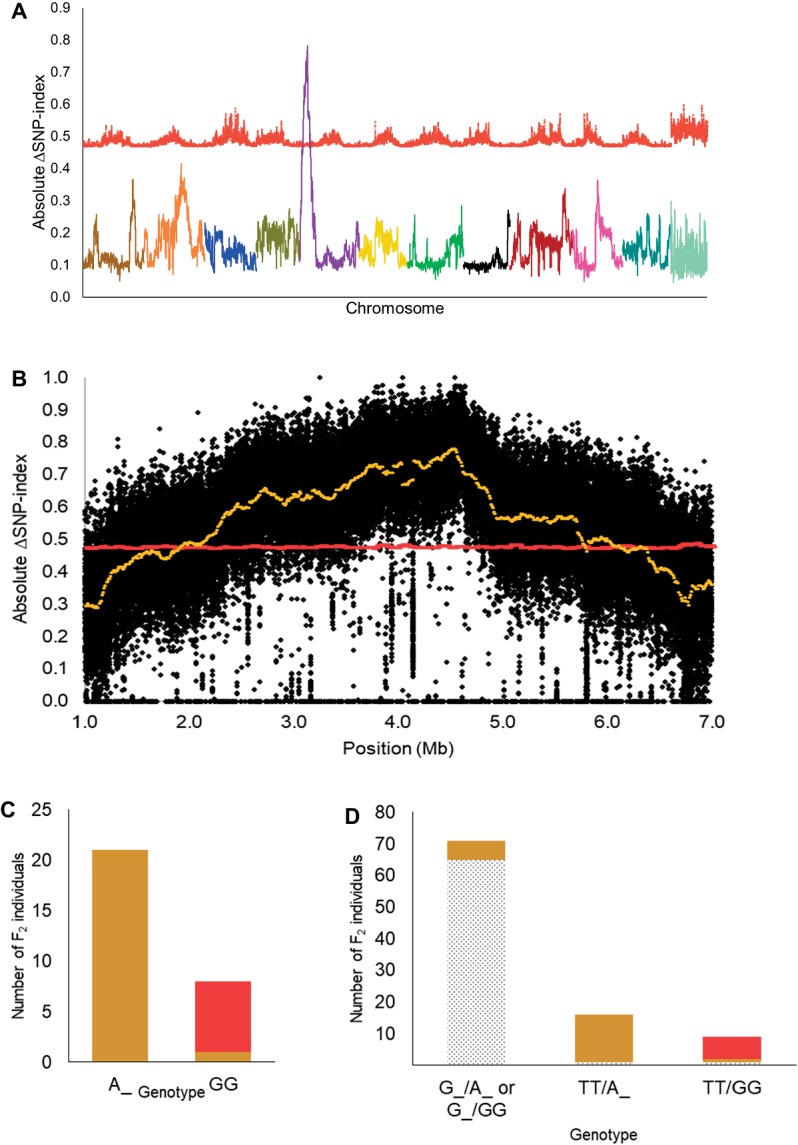
**(A)** Mapping of the *T^1^* locus as a significant peak on chromosome 5 using QTL-seq. Absolute ΔSNP-index of all chromosomes, obtained by subtracting SNP-index of the red bulks from the tan^1^ bulk in the dotted black × red population, is plotted along with statistical confidence intervals under the null hypothesis of no QTL (*p* = 0.01) (red line). Chromosomes are aligned in sequential order from 1 to 11 and 0. **(B)** Magnified view of significant ΔSNP-index peak (yellow) associated with *T^1^* locus along with absolute ΔSNP-index of SNPs (black circles) plotted against SNP position based on 97103 watermelon genome (Guo et al., 2013). **(C)** Association of KASP™ marker UGA5_4591722 with the tan^1^ and red seed coat phenotype in the dotted black × red F_2_ population (*n* = 29). The *x*-axis denotes the genotype of F_2_ individuals for KASP™ marker UGA5_4591722 and the *y*-axis denotes the number of F_2_ individuals with tan^1^ (tan bar) and red seed coat color (red bar). **(D)** Bar graph indicating the phenotypic prediction accuracy of KASP™ markers UGA3_5820134 and UGA5_4591722 in the dotted black × red population (*n* = 96). The genotypes on the *x*-axis represent the alleles of the UGA3_5820134/UGA5_4591722 markers. The dotted black, tan, and red sections in the graph indicate the number of F_2_ individuals with respective seed coat color.

Based on the 12:3:1 ratio associated with dominant epistasis, we inferred that the tan^1^ seed coat color and the red seed coat color were segregating for a single gene. Therefore, we pooled DNA from individuals with the tan^1^ seed coat color and the red seed coat color to develop the T-bulk and the R-bulk, respectively. From QTL-seq, a significant ΔSNP-index peak (*p* < 0.01) was mapped from 1.89 to 6.46 Mb on chromosome 5 of the *C. lanatus* genome (Figures 3A,B). A SNP present within a significant peak and positioned at 4,591,722 bp on chromosome 5 of the 97103 reference genome (Guo et al., 2013) was utilized to develop the UGA5_4591722 KASP™ assay (Table 1) to test the association of the peak and the phenotype. The marker was able to predict tan^1^ (genotype: A:A or G:A) or red seed color (genotype: G:G) with 96.55% accuracy (*n* = 29) validating that the peak is related to the seed coat color (Figure 3C). Since the region mapped in this population was different from the region mapped in the dotted black × green population, we concluded that this region is not the *R* locus and based on the nature of inheritance, it can be inferred that this region is either a novel locus or a different allele of the *T* locus described by Poole et al. (1941). Therefore, we propose to name this locus *T^1^*, in conformance with gene nomenclature rules for Cucurbitaceae (Cucurbit Gene List Committee, 1982).

We also tested the KASP™ assay UGA3_5820134 associated with the *R* locus and found that the dotted black × red population was segregating for the *R* locus, as predicted by the four-gene model. Approximately 97.01% of individuals with dotted black seed color had the genotype G:G or T:G and 79.31% of individuals with tan^1^ or red seed color had the genotype T:T (Supplementary Figure S2). In addition, the genotypic data from the combination of KASP™ markers UGA3_5820134 and UGA5_4591722 were analyzed to understand the interaction between the two loci. Out of 71 F_2_ individuals that had the genotype G:G or T:G for marker UGA3_5820134, 65 individuals (91.54%) had dotted black seed coat color, independent of the UGA5_4591722 genotype (Figure 3D). Among 16 F_2_ individuals that had the genotype T:T for marker UGA3_5820134 and A:A or G:A for marker UGA5_4591722, 15 individuals (93.75%) had tan^1^ seed coat color. Similarly out of 9 F_2_ individuals that had the genotype T:T for marker UGA3_5820134 and G:G for marker UGA5_4591722, 7 individuals (77.77%) had red seed coat color. This confirms our hypothesis that the *R* locus is dominantly epistatic to *T^1^* locus.

### Phenotypic Segregation in the Dotted Black × Clump Population and Mapping of the *W* and *D* Loci

Based on the four-gene model, the dotted black genotype (*RTWd*) and the clump genotype (*RTwD* or *RTwd*) segregate either for the *W* gene or for both *W* and *D* genes. In the dotted black × clump population, the F_1_ had flat black seed coat color (*W_D_*) meaning that the genotype of the clump parent, UGA147, is expected to be *RRTTwwDD* and the population is segregating for both the *W* and *D* genes (Figure 1C). The F_2_ progenies segregated as flat black (*W_D_*, *n* = 94), dotted black (*W_dd*, *n* = 35), and clump (*wwD_* or *wwdd*, *n* = 49) which statistically fits a 9:3:4 ratio ($\chi_{\left(0.05,2\right)}^{2}$ = 0.91, *p* = 0.63), confirming the conclusion by Poole et al. (1941) that the *D* gene is recessively epistatic to *W*.

**Figure 4:**
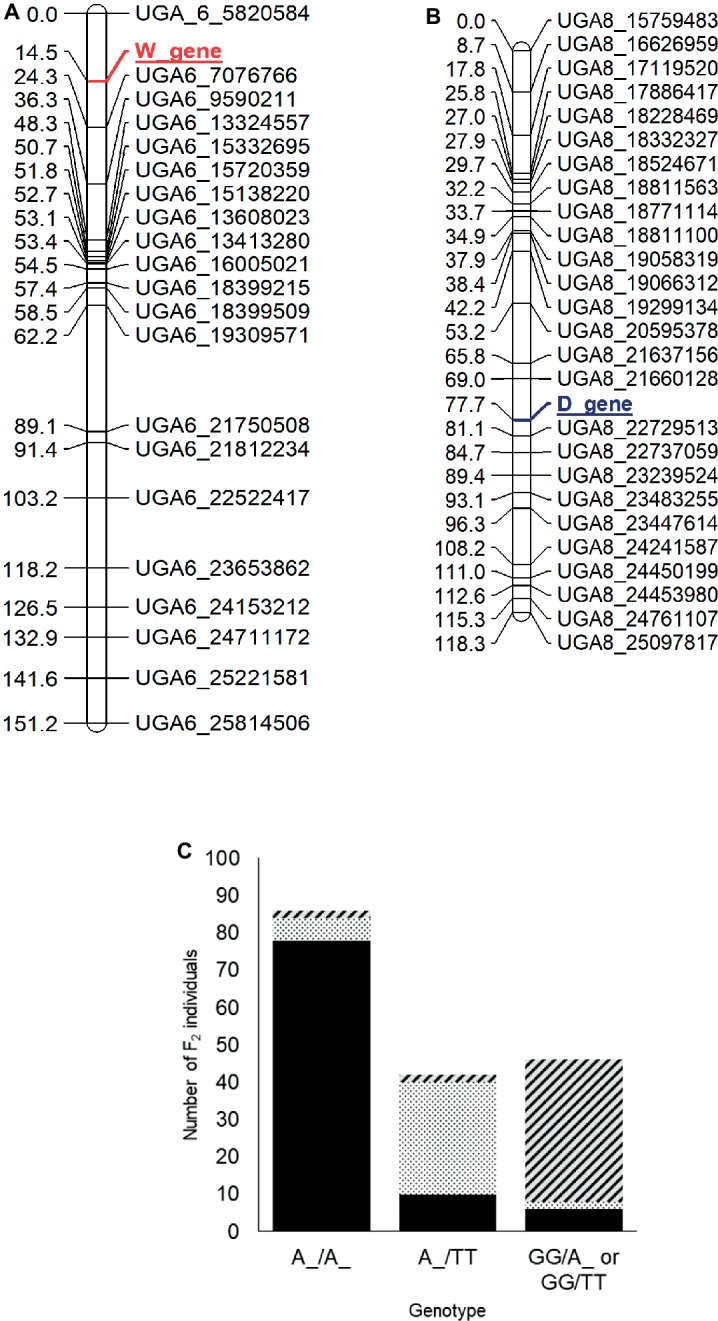
Genetic maps of chromosome 6 **(A)** and 8 **(B)** showing the position of the *W* and *D* loci, respectively, in the dotted black × clump F_2_ population developed from a cross between dotted black seeded Charleston Gray and clump seeded UGA147 (selection from PI 169233). The marker names indicate the chromosome number and physical position of the marker based on the 97103 watermelon genome (Guo et al., 2013). **(C)** Bar graph indicating phenotypic prediction accuracy of SNP markers UGA6_7076766 and UGA8_22729513 in the dotted black × clump population (*n* = 174). The genotype on the *x*-axis represents the alleles of markers UGA6_7076766/UGA8_22729513 in the F_2_ population. The sections with black, dotted black, and diagonal black lines in the graph indicate number of F_2_ individuals with flat black, dotted black and clump seed coat color, respectively.

For mapping of the *W* and *D* genes, the seed phenotypes were translated into the “*abhcd*” genotype code format as described in the Joinmap® 4 manual (Van Ooijen, 2006). For the *W* locus, all individuals with non-clump seed coat color (flat black and dotted black, genotype: *W_*) were coded *d* (not clump parent genotype) and individuals with clump seed coat color (genotype: *ww*) were coded *b* (clump parent genotype). Similarly, for the *D* locus, all the individuals with flat black seed color (genotype: *D_*) were coded *c* (not dotted black parent genotype), and individuals with dotted black seed coat color (genotype: *dd*) were coded *a* (dotted black parent genotype). Individuals with the clump phenotype (genotype: *D_* or *dd*) were coded as missing data since the genotype of clump seeded individuals could not be predicted from the F_2_ phenotype. The two phenotypic markers along with 230 SNP markers were used to construct a genetic map. Thirteen linkage groups with a total length of 1,226 cM and an average marker distance of 5.3 cM were developed for the 11 watermelon chromosomes (Supplementary Figure S3). The *W* locus was mapped at 14.5 cM on chromosome 6 between markers UGA6_5820584 and UGA6_7076766 (Figure 4A). The closest marker UGA6_7076766 is 9.8 cM away from the *W* locus. The genomic region associated with *W* locus partially overlapped with the major seed length QTL, *Qsl6^M^* (Prothro et al., 2012; Meru and McGregor, 2013). This is in accordance with the conclusion by Poole et al. (1941) that the *W* locus is linked with the *L* locus associated with seed length. The *D* locus was mapped between markers UGA8_21660128 and UGA8_22729513 at position 77.7 cM on chromosome 8 on the dotted black × clump genetic map (Figure 4B). The closest marker, UGA8_22729513 is 3.4 cM away from the *D* locus.

We analyzed the genotypic data of SNP markers UGA6_7076766 and UGA8_22729513 to examine if the combination of *W* and *D* loci could predict seed coat color. Whenever F_2_ individuals were homozygous dominant or heterozygous for both *W* (A:A or A:G genotype for marker UGA6_7076766) and *D* locus (A:A or A:T genotype for marker UGA8_22729513), 90.69% of individuals had flat black seed color (Figure 4C). Similarly, when F_2_ individuals were homozygous dominant or heterozygous for *W* locus but recessive for the *D* locus (T:T genotype for marker UGA8_22729513), 71.42% individuals had dotted black seed coat color. However, when F_2_ individuals were homozygous recessive for the *W* locus, (G:G genotype for marker UGA6_7076766), 82.60% of individuals had clump seed color irrespective of the *D* locus. In total, the phenotypic prediction accuracy of markers UGA6_7076766 and UGA8_22729513 when used as a proxy for *W* and *D* loci was 82.60% (*n* = 174). The percentage of inaccurate phenotype prediction (17.40%) is similar to the value of total recombination between SNP markers and respective loci (13.2 cM). Our result confirms that the genomic regions identified on chromosome 6 and 8 are associated with the *W* and *D* locus, respectively, and that the *D* locus is recessively epistatic to the *W* locus.

## Discussion

Genetic mapping of traits has always been a subject of interest to plant breeders. With the advent of NGS, the genotyping process has become fast, highly accurate, and relatively cheap. However, phenotyping still remains as a major bottleneck for efficient mapping of genetic traits. In watermelon, seed coat color can be phenotyped in major categories through visual analysis. Within these broad phenotypic categories described by Poole et al. (1941) and used in this study, other subtler variation was observed. This variation could be attributed to both genetic and non-genetic factors. One of the most important factors is the maturity of seeds. In our study, we found that maturity creates different variations in the phenotype. The number and size of black dots and the background color (color beneath black dots) of the dotted black seeds varied not only among individuals but also within the same individual, indicating that at least some of the variation within this phenotype can be attributed to non-genetic factors. Less mature seeds usually had very few fine dots on light brown background whereas mature seeds had many large dots on dark brown background. The individuals with green seed color also had different shades of green ranging from light green to dark green to brownish green depending upon the maturity of the seeds. Within the same individual, more mature fruit had brownish green seed color, while less mature fruit had light green seeds (Supplementary Figure S1, Figure 1A). Maturity has also been identified as one of the non-genetic factors to influence phenotype by Poole et al. (1941). One of the possible ways to avoid effects of maturity would be to phenotype seeds of the same maturity stage. Avoiding the effect of maturity could possibly allow the use of quantitative measurements to phenotype seed coat color. However, in the current study, the *C. lanatus* × *C. amarus* populations were also segregating for maturity, leading to different F_2_ progenies’ fruits maturing at different rates. Fruits of early maturing individuals mature relatively sooner, and the flesh of those fruits starts to ferment and affect the phenotype (Poole et al., 1941). Whereas, in late maturing individuals, seeds are immature and not showing the mature phenotype. Additional research to develop a better phenotyping method which can avoid the effect of non-genetic factors in phenotyping is essential to understand the subtler phenotypes and identify additional loci involved in the genetics of watermelon seed coat color.

The four-gene model was developed by Poole et al. (1941) based on the inheritance of seed coat color in their populations and the populations developed by McKay (1936). Since the development of the four-gene model, only a few studies have been conducted to study inheritance of seed coat color. Nath and Dutta (1973) crossed a tan seeded individual with a red seeded individual and found that tan is monogenically dominant to red as predicted by the model. Similar studies by Sachan and Nath (1976) and Nath and Khandelwal (1978), crossing flat black seeded and tan (referred to as brown in the study) seeded individuals, also fit the four-gene model. However, the model was contradicted in a cross made by Nath and Khandelwal (1978) where flat black seed coat color was monogenically dominant to red seed coat color. In a similar study conducted by Sharma and Choudhury (1982), they found that flat black seed coat color and white seed color segregate only for one gene instead of two or three genes (depending on whether white seed is white-tan tip or white-pink tip) as predicted by the four-gene model. In the current study, the inheritance of the *R*, *W* and *D* loci fit the four-gene model, however, inheritance of the *T^1^* locus did not. The *T^1^* locus mapped in the dotted black × red population could be a different allele of the *T* locus or even a novel gene. Further testing of allelism is hampered by the lack of information about the identity of the red parental genotype used in the study by McKay (1936), which was used by Poole et al. (1941) in developing the four-gene model. The genotype is simply described as “citron,” which is equivalent to *C. amarus*, but no further information is provided. The parental genotypes, “Peerless” and “Baby Delight,” which produced red phenotype when crossed in Poole et al. (1941), are not currently available to replicate the cross. Nevertheless, findings from the current study and several others demonstrate that the four-gene model is incomplete and requires amendment.

Seed coat color is a complicated trait not only because of the number of genes involved in conferring phenotype but also because of the interactions among these loci. Understanding inheritance of the seed coat color phenotypes, the genetics and interaction of the different genes involved, identifying new genes, allelic variations and interactions among them requires developing, phenotyping and genotyping many populations. This is an arduous task to be done in one study because of the time, labor, and cost involved. The easier solution for this is to analyze and compare results of several studies and derive a consensus conclusion. However, the lack of the standard phenotypic descriptors makes it difficult to do so. In each study, the authors develop their own phenotyping methodology which makes it difficult to compare results among experiments (Weetman, 1937; Poole et al., 1941; Sachan and Nath, 1976; Nath and Khandelwal, 1978). This has been exacerbated by the fact that some of the lines/cultivars used in previous studies are no longer available to replicate the crosses. Since the phenotypic description developed by Poole et al. (1941) is the most detailed among any studies previously conducted, we propose that future studies related to seed coat color in watermelon should use the phenotypic description developed by Poole et al. (1941). Any new phenotypic class like tan^1^ should only be used if it is distinct from the previously developed class or has a different inheritance pattern.

## Conclusion

To conclude, this is the first study to map seed color gene loci in watermelon and to report SNP markers associated with these loci. Most of the prior research related to the genetics of watermelon seed color was carried out before the advent of molecular tools. In this study, we mapped the *R*, *T^1^*, *W* and *D* loci on chromosomes 3, 5, 6 and 8, respectively, and developed markers UGA3_5820134, UGA5_4591722, UGA6_7076766, and UGA8_22729513 for MAS of seed coat color in watermelon. Further research is necessary to determine whether *T^1^* is a different allele or different locus than the previously described *T* locus. Moreover, identification of the *T^1^* locus indicates that there are additional genes/alleles that confer seed coat color in watermelon. Our results also open future research opportunities to fine map genomic regions and identify the genes conferring seed coat color and to identify functional markers for MAS of seed coat color in watermelon.

## Data Availability

The raw data supporting the conclusions of this manuscript will be made available by the authors, without undue reservation, to any qualified researcher.

## Author Contributions

LP conducted research and wrote the manuscript as a part of his MS research. JC guided LP on analysis of QTL-seq data. CM conceived the project, guided research and data analysis, and revised the manuscript before submission.

### Conflict of Interest Statement

The authors declare that the research was conducted in the absence of any commercial or financial relationships that could be construed as a potential conflict of interest.

## Abbreviations

MAS: Marker-assisted selection
KASP™: Kompetitive Allele Specific PCR
NGS: Next generation sequencing
GBS: Genotyping-by-sequencing
PI: Plant Introduction.
