# Supplementary material for: Chromosomal Locations and Interactions of Four Loci Associated With Seed Coat Color in Watermelon
Source: Front Plant Sci. 2019 Jun 25;10:788. doi: 10.3389/fpls.2019.00788 (PMC6603093; doi:10.3389/fpls.2019.00788)
Supplement: Supplementary file 1 [file Table_1.DOCX]

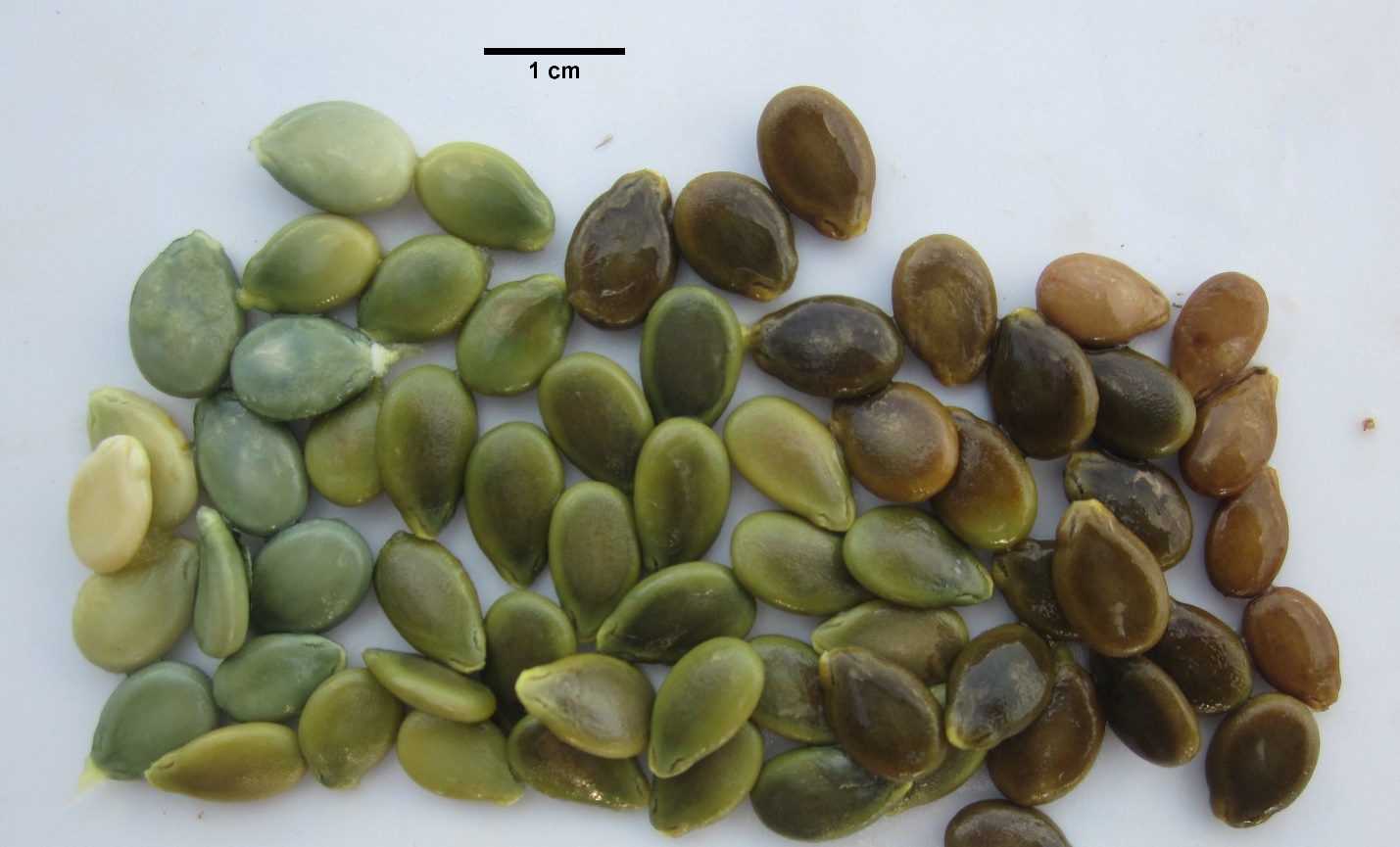


**Supplementary Figure S1.** Non-genetic variation in green seed coat color phenotype. All seeds were harvested from fruits of a single F_2_ plant in the dotted black (Sugar Baby) x green (PI 482379) population.

**Supplementary Figure S2.** Phenotypic prediction accuracy of KASP^TM^ markers UGA3_5820134 and in the dotted black x red population (n = 96). The genotypes on the x-axis represents the alleles of the UGA3_5820134 marker, and the dotted black, tan and red sections in the graph indicate the number of F_2_ individuals with respective seed coat color

**Supplementary Figure S3.** A genetic linkage map of the dotted black x Clump F_2_ population developed from a cross between a dotted black seeded Charleston Gray and a clump seeded UGA147, selection from PI 169233. 230 SNP markers and 2 phenotypic markers present on 11 watermelon chromosomes have been grouped into 13 linkage groups which spans 1226 cM. The marker names are indicated in the left side of the bars and genetic distance in cM is indicated in the right side of the bars
